# Molecular mechanisms of ferroptosis and the potential therapeutic targets of ferroptosis signaling pathways for glioblastoma

**DOI:** 10.3389/fphar.2022.1071897

**Published:** 2022-11-24

**Authors:** Meng Zhang, Qian Lei, Xiaobo Huang, Yi Wang

**Affiliations:** ^1^ Department of Anesthesiology, Sichuan Academy of Medical Science and Sichuan Provincial People’s Hospital, University of Electronic Science and Technology of China, Chengdu, China; ^2^ Department of Critical Care Medicine, Sichuan Academy of Medical Science and Sichuan Provincial People’s Hospital, University of Electronic Science and Technology of China, Chengdu, China

**Keywords:** ferroptosis, glioblastoma, ROS, GPx4, iron metabolism

## Abstract

Ferroptosis is a newly identified form of cell death that differs from autophagy, apoptosis and necrosis, and its molecular characteristics include iron-dependent lipid reactive oxygen species accumulation, mitochondrial morphology changes, and membrane permeability damage. These characteristics are closely related to various human diseases, especially tumors of the nervous system. Glioblastoma is the most common primary malignant tumor of the adult central nervous system, and the 5-year survival rate is only 4%–5%. This study reviewed the role and mechanism of ferroptosis in glioblastoma and the research status and progress on ferroptosis as a potential therapeutic target. The mechanism of ferroptosis is related to the intracellular iron metabolism level, lipid peroxide content and glutathione peroxidase 4 activity. It is worth exploring how ferroptosis can be applied in disease treatment; however, the relation between ferroptosis and other apoptosis methods is poorly understood and methods of applying ferroptosis to drug-resistant tumors are insufficient. Ferroptosis is a promising therapeutic target for glioblastoma. In-depth studies of its mechanism of action in glioblastoma and applications for clinical treatment are expected to provide insights for glioblastoma patients.

## Introduction

Glioblastoma (GBM) is a common primary intracranial tumor that has a poor prognosis, not only because of abnormal cell proliferation but also because of tumor infiltration into normal brain tissue and resistance to radiotherapy (Liu et al., 2015; Wirsching et al., 2016). For such a difficult tumor, there is an urgent need to develop efficient and specific treatment tools. GBM cells take advantage of their tumor microenvironment to produce intracellular and extracellular homeostasis to escape or reduce cell death (Xiao et al., 2019; De Biase et al., 2021). Regulated cell death (RCD) is a genetically regulated death process initiated by the intracellular or extracellular microenvironment when other adaptive responses fail to restore intracellular and extracellular homeostasis (Chen et al., 2021; Shan et al., 2022). Among cell death processes, ferroptosis is a newly discovered nonapoptotic modality that is iron-dependent and mediated by the accumulation of reactive oxygen species (ROS), particularly lipid peroxides, which accumulate in large amounts and cause an intracellular redox imbalance that leads to ferroptosis of the cells (Hong et al., 2021; Wang et al., 2022).

Ferroptosis has been shown to play an important role in neurological tumor diseases, and it represents a unique death form with various biological characteristics. The main characteristic manifestation of ferroptosis is the continuous accumulation of ROS. Due to the activation of the original oncogene in GBM cells, they produce ROS (Luo et al., 2021a; Hong et al., 2021). Moreover, the rapid proliferation of tumor cells requires abundant nutrients and energy, and these cells then undergo metabolic reprogramming as glycolysis. Meanwhile, abnormal mitochondrial function, which produces more ROS, is also found in proliferating cancer cells (de Lucas et al., 2016; Zhang et al., 2020a). At a certain ROS level, proliferative signals are conducted and GBM development is promoted (Ansari et al., 2017; Larson et al., 2022). However, excessive ROS also causes cell death; therefore, GBM cells need to raise their antioxidant levels to maintain the redox balance. Recent research revealed that ferroptosis play critical role in GBM progression. Inducing ferroptosis leads to suppressed growth of GBM cells accompanied with mitochondrial dysfunction (Shi et al., 2014; Miki et al., 2022). Decreased ferroptosis related markers has been correlated with poor prognosis and relapse of GBM patients (Dong et al., 2022; Kram et al., 2022; Zhong et al., 2022). Furthermore, inducing ferroptosis could enhance the drug sensitivity of GBM (Mou et al., 2019; Mitre et al., 2022). Therefore, it is of crucial importance to summarize the mechanisms of ferroptosis and the relationship between ferroptosis and glioblastoma.

## Molecular mechanisms of ferroptosis

**FIGURE 1:**
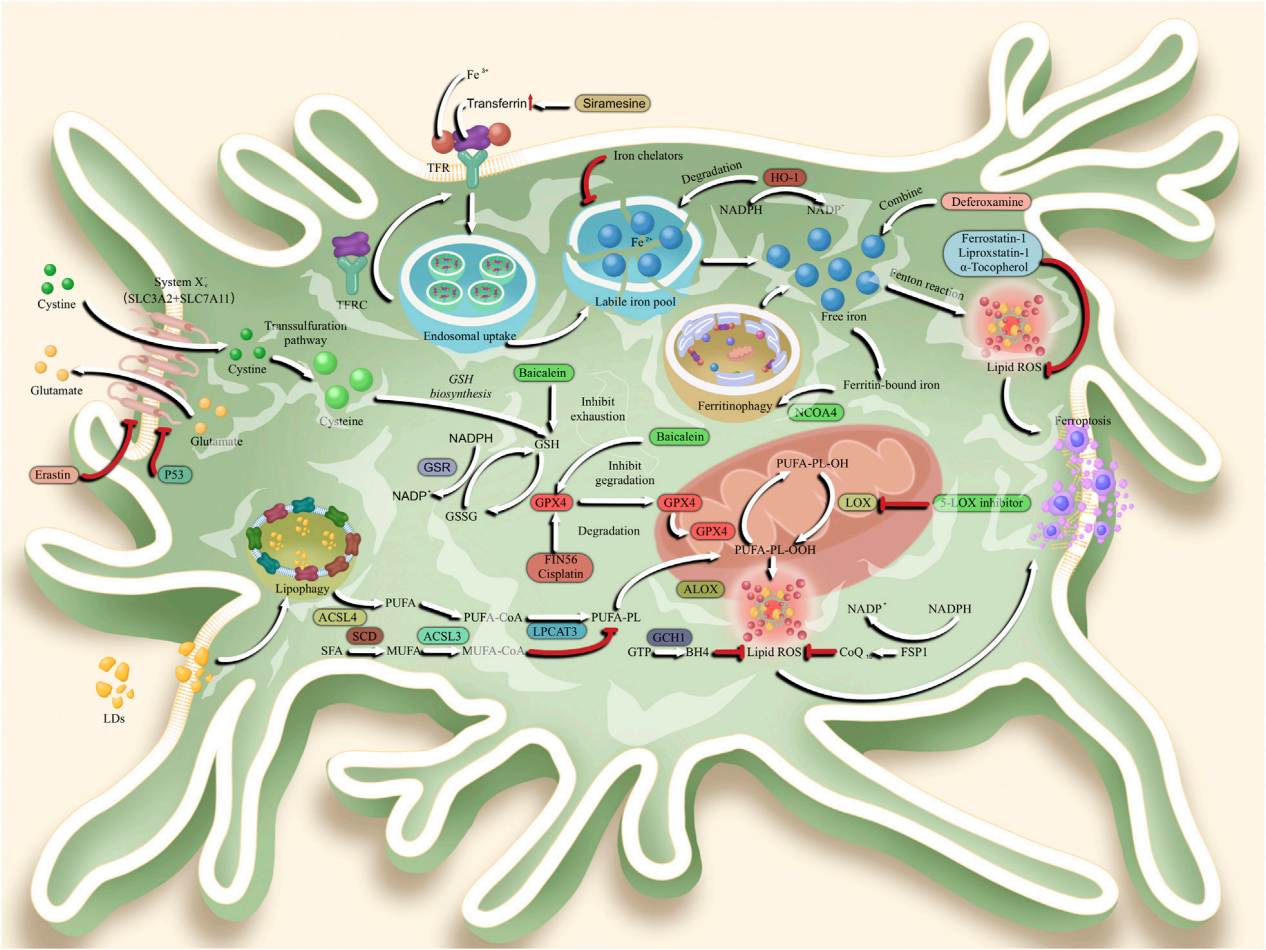
Mechanisms of ferroptosis in glioblastoma, In the ferroptosis signaling pathway, System Xc-transports cystine to the cytoplasm of glioblastoma multiforme (GBM) cells. System Xc-is composed by two subunits, which are solute carrier family 7 member 11 (SLC7A11) and solute carrier family 3 member 2 (SLC3A2). Subsequently, cystine was degraded to cysteine for glutathione (GSH) biosynthesis. Followed that, glutathione peroxidase 4 (GPX4) enters the mitochondria to reduce the toxicity of lipid reactive oxygen species (ROS) and maintain the homeostasis of membrane lipid bilayer homeostasis. In another ferroptosis pathway, transferrin combined with transferrin receptor (TRF) to shift Fe3+ into cytoplasm. Followed by endosomal uptake, Fe3+ was transported to labile iron pool as a form of Fe2+. When degraded by Heme oxygenase 1 (HO-1), free iron is released to stimulate lipid ROS generation and ferroptosis by Feton reaction. The Ferritin-bound iron also undergo ferritinophagy *via* nuclear receptor coactivator 4 (NCOA4). Meanwhile, lipid drops (LDs) will enter the cytoplasm *via* lipophagy, and transform polyunsaturated fatty acid (PUFA) into PUFA-Coenzyme A (PUFA-CoA) *via* acyl-CoA synthetase long chain family member 4 (ACSL4). Under the esterification of lysophosphatidylcholine acyltransferase 3 (LPCAT3), PUFA was further transformed into PUFA-Phospholipids (PUFA-PL). Then, PUFA-PL is transported to mitochondria and are over-oxidized to toxic lipid peroxides (PL-PUFA-OOH), a process that relies on free radicals provided by the Fenton reaction. Subsequently, lipid ROS is generated to stimulate the ferroptosis.

Ferroptosis is a newly discovered programmed cell death process that is morphologically manifested by outer mitochondrial membrane rupture, increased inner mitochondrial membrane density, mitochondrial cristae reduction or disappearance, normal nuclei and no chromosome condensation (Clemente et al., 2020; Zhou et al., 2022). In terms of metabolic changes, they mainly exhibited iron deposition, lipid peroxidation damage to the cell membrane, and decreased glutathione (GSH) content (Figure 1) (Amic et al., 2021; Meyer et al., 2021). At present, the detection of ferroptosis in cells is also based on the above morphological and metabolic changes to cells, which can be determined by electron microscopy, tissue iron content changes, and intracellular oxidative stress-related indices, such as ROS, MDA content and GSH changes (Diaz-Vivancos et al., 2015; Park and Chung, 2019).

### Glutathione peroxidase 4 inactivation or depletion induces ferroptosis

Glutathione peroxidase 4 (GPX4) scavenges lipid peroxides to generate lipid alcohols, which inhibits Fe^2+^ induced reactive oxygen species (ROS) production (Shyu et al., 2014; He et al., 2020). It can disrupt the oxidation balance, with the accumulated lipid peroxides promoting the destruction of the membrane structure and stimulating ferroptosis. Therefore, GPX4 reduces membrane lipid hydrogen peroxide (LOOH) to a nontoxic lipid alcohol (LOH) by consuming the prototype glutathione enzyme (Zhou et al., 2018; Tian et al., 2022). Since GPX4 mainly utilizes the antioxidant glutathione to reduce lipid hydrogen peroxide, GPX4 activity is largely dependent on intracellular GSH levels (Liu et al., 2019a; Luo et al., 2021b). Inhibition of GPX4 activity leads to excessive lipid peroxidation and subsequent cell ferroptosis; for example, RSL3 GPX4 inhibitors can induce cell ferroptosis by reducing GPX4 expression (Hu and Liu, 2020; Zhou et al., 2022). Despite the crucial role of GPX4 in ferroptosis, certain cancer cells are resistant to ferroptosis caused by GPX4 inhibitors, suggesting that ferroptosis may also be regulated by other factors. Reports have shown that ferroptosis can be activated by small molecules, such as erastin, although inhibition of GPX4 activity is the primary factor because it increases the production of intracellular ROS, thereby promoting intracellular lipid peroxidation and inducing ferroptosis (Battaglia et al., 2020; Kram et al., 2022).

### Inhibition of system Xc^−^-GSH-GPX4 activity induces ferroptosis

Cysteine/glutamate reverse transporter (system Xc^−^) is located on the cell membrane and mainly loaded by SLC3A2 and SLC7A11 (Aldape et al., 2015; Zhong et al., 2022). System Xc^−^ transports amino acids in a ratio of 1:1 by pumping in extracellular cystine while pumping out intracellular glutamate. The ingested cystine undergoes an enzymatic reaction to form cysteine (Legendre et al., 2016; Yang et al., 2018). Cysteine synthesizes γ-glutamine cysteine under ATP-dependent cysteine-glutamate ligase, which synthesizes glutathione (GSH) by glutathione synthetase (Pobee et al., 2021; Dong et al., 2022). GSH is a key factor regulating ferroptosis and is responsible for scavenging free radicals, and it is also an important antioxidant in the human body. Thus, when system Xc-induced uptake of cystine is inhibited, GSH synthesis is also inhibited, thus causing peroxide accumulation and inducing ferroptosis (Zhang et al., 2020b; Zhang et al., 2021a). Cysteine can limit glutathione biosynthesis. When cells are in a reducing environment, cysteine can be directly transported into cells through the alanine-serine-cysteine (ASC) system to inhibit ferroptosis (Reinert et al., 2019; Li et al., 2020a). In addition, cystathionine is another source of cysteine, and cells use the reverse transsulfuration pathway to synthesize cystathionine from methionine, which is converted to cysteine. Synthetic GSH acts as an electron donor under the action of GPX4 to convert phospholipid peroxides to phospholipid alcohols, and oxidized glutathione, hexamethyl melamine or GPX4 expression-induced genetic interference can induce ferroptosis (Xiao et al., 2015; Saalik et al., 2019).

### Induction of lipid ROS generation

Intracellular ROS include superoxide anions, hydrogen peroxide, and hydroxyl radicals. During oxidative phosphorylation, mitochondria produce ATP, and electron leakage during electron transport or NADPH oxidase on the cell membrane leads to the production of superoxide anions. Under the action of superoxide dismutase, these anions form H_2_O_2_, which is catalyzed by metal elements, such as iron, to produce hydroxyl radicals, thereby inducing lipid peroxidation and leading to ferroptosis (Srivastava et al., 2018; Tian et al., 2022). In tumor cells, cells produce ROS due to the activation of proto-oncogenes (Yu et al., 2018; Jiang et al., 2022). Studies have shown that a certain ROS level can conduct proliferative signals and promote tumor development. However, excessive ROS also cause cell death; therefore, the antioxidant levels of tumor cells must be increased to maintain the redox balance (Khuong-Quang et al., 2012; de Lucas et al., 2016). Iron is an important cofactor in metabolically active cells, such as neurons. Mitochondrial function, axonal transport and myelin formation require the fine regulation of iron (Liu et al., 2019b; Nakamura et al., 2019). When the cystine glutamate reverse transporter is inhibited, intracellular glutathione (GSH) synthesis is reduced and GPX4 function is inhibited, which leads to the production of large amounts of intracellular lipid ROS (Deane et al., 2004; Yu et al., 2021). However, intracellular ferrous ions can be associated with intracellular ROS *via* the Fenton reaction. During ROS binding, a large amount of lipid ROS with extremely strong oxidative activity is generated, which eventually leads to ferroptosis (Zhang et al., 1996; Kang et al., 2020).

## Ferroptosis in glioblastoma

Ferrostatin-1, a ferroptosis inhibitor, acts as a free radical trapping antioxidant that inhibits intracellular ROS production and effectively suppresses glutamate-induced cell death in the mouse hippocampus. Treatment with ferrostatin-1, a ferroptosis inhibitor, significantly inhibits oxidative stress in brain tissue, improves brain tissue structure after hemorrhage, protects neurons, inhibits glial scar formation, and promotes glial scar formation in rats (Legendre and Garcion, 2015; Liu and Gu, 2022). Erastin is a small molecule ferroptosis inducer that can induce ferroptosis by inhibiting the cystine-glutamate transport pathway, activating the p53 gene pathway, and eliminating the inhibitory pathway of microtubulin on voltage-dependent anion channels (VDACs) (Kuang et al., 2014; Yu et al., 2020). Compared with other ferroptosis inducers, erastin has the advantages of high efficiency, rapidity and long duration of action (Dai et al., 2020; Zhang et al., 2021b). The molecular mechanisms of ferroptosis described above indicate that multiple pathways are involved in its regulation (Li et al., 2020b). We summarize the relationship between certain pathways and glioblastoma and reveal their potential effects on glioblastoma (Sang et al., 2019; Tsai et al., 2021).

### Crucial role of transferrin in ferroptosis-mediated GBM

In recent years, an increasing number of studies have begun to explore the mechanisms of iron action in the nervous system (Wang et al., 2020; Su et al., 2021). Studies have identified transferrin receptors on the surface of brain capillaries. Dysregulation of transferrin (Tf) metabolism leads to an increase in unstable iron in the brain, which in turn leads to lipid peroxidation. Indeed, sufficient unstable iron in the blood is necessary for the generation of ferroptosis (Park et al., 2020; Wei et al., 2020). Another finding shows that antioxidants reduce the content of milk iron and inhibit the production of iron-dependent lipid ROS. Transferrin expression is much higher in GBM than in other grades of glioma, and 8-hydroxydeoxyguanosine (8-OHd G), which indicates the oxidative stress level, showed the highest expression, thus indicating a positive correlation between the degree of tumor cell malignancy and the occurrence of cellular ferroptosis (Lei et al., 2020; Garcia-Gomez et al., 2022). Tf is an 80 k Da glycoprotein found in plasma and represents an essential growth factor for cell proliferation (Ratan, 2020; Zhao et al., 2022). However, Tf is not present in all brain regions but mainly in oligodendrocytes, cerebrospinal fluid and brain capillary endothelial cells (Libby et al., 2021; Liu et al., 2022). Regulation of Tf is accomplished through a specific set of proteins that regulate the concentration of iron in tumor cells (Li et al., 2014; Yang et al., 2022).

### Crucial role of amino acid metabolism in ferroptosis-mediated GBM

Activation of the NRF2 pathway in glioblastoma can inhibit ferroptosis by upregulating system Xc^−^. Transcriptional activator 4 enhances the transcription of system Xc^−^, thereby inhibiting ferroptosis in glioblastoma cells (Li et al., 2014; Song et al., 2021). Silencing of system Xc^−^ enhances the sensitivity of glioma cells to the chemotherapeutic agent temozolomid (Wu et al., 2019; Zhang et al., 2021c). Tulipic acid induces ferroptosis in glioblastoma cells by activating NADPH oxidases 4 (NOX4) and inhibiting system Xc^−^. The ferroptosis inducer erastin in combination with temozolomide enhances the killing effect of temozolomide on glioblastoma cells by inhibiting system Xc^−^. The cystine/glutamate anti-transporter (system Xc^−^) takes in cystine at a 1:1 ratio on the plasma membrane, excretes glutamate, and reduces the cystine taken up into cells to cysteine (Yao et al., 2021; Xu et al., 2022). Then, cysteine is combined with glutamate and glycine to form glutathione (glutathione, GSH), which is catalyzed by GPX4 and oxidized to produce a dynamic balance. Inhibition of GPX4 and GSH synthesis initiates ferroptosis, and inhibition of cysteine uptake leads to lipid peroxide accumulation (Zhang et al., 2021a; Ma et al., 2022). The ferroptosis inducer erastin can also lead to ferroptosis through the amino acid metabolism pathway (that is, inhibiting system Xc^−^ to reduce intracellular cysteine and GSH, thereby inhibiting GPX4 from clearing ROS) (Yi et al., 2020; Gao et al., 2022).

### Association of ferroptosis-related genes in GBM

Ferroptosis is regulated by genes and leads to various metabolic changes (Zhang et al., 2020c; Li et al., 2021a). According to different protein metabolism pathways, ferroptosis-related genes are divided into four categories: genes related to iron metabolism, which include ACO1 (aconitase 1), CISD1 (CDGSH iron-sulfur domain-containing protein 1, also termed mitoNEET), FANCD2 (FA complementation group D2), FTH1 (ferritin heavy chain 1), HMOX1 (heme oxygenase 1), HSBP1 (heat shock factor binding protein 1), IREB2 (iron responsive element binding protein 2), NCOA4 (nuclear receptor coactivator 4), PHKG2 (phosphorylase kinase catalytic subunit gamma 2), STEAP3 (six transmembrane epithelial antigen of the prostate 3), TFRC (transferrin receptor); genes related to lipid metabolism, which include ACSF2(Acyl-CoA synthetase family member 2), ACSL4 (Acyl-CoA synthetase long-chain family member4), GPX4, LPCAT3 (lysophosphatidylcholine acyltransferase 3), PEBP1 (phosphatidylethanolamine binding protein 1); genes related to antioxidant metabolism, which include MRP1 (material requirements planning), GCLC (glutamate-cysteine ligase catalytic subunit), HMOX1 (Heme oxygenase-1), KEAP1 (kelch-like ECH associated protein1), NQO1 (NADPH quinone oxidoreductase1), NRF2 (nuclear factor erythroid 2-related factor 2), SLC7A11 (solute carrier family 7 member 11), G6PD (glucose-6-phosphatedehydrogenase), GLS2 (glutaminase 2), GOT1(glutamic-oxaloacetic transaminase 1), SLC1A5 (solute carrier family 1 member 5) (Dong et al., 2020).

The expression levels of the ferroptosis-related lncRNAs PVT1 and GLUT3 are upregulated in GBM, and their expression levels are significantly positively correlated. Patients with high expression of the two have a lower overall survival rate (Zheng et al., 2016). PVT1 may regulate the expression of GLUT3 through miR-29b-3p (Fu et al., 2021). Promoting the malignant progression of glioma, PVT1 can also regulate ferroptosis during cerebral ischemia/reperfusion through miR-214-mediated TFR1 and p53(88). We also found that miR147a stimulates ferroptosis in glioblastoma by targeting SLC40A1. miR-18a has been reported to directly target ALOXE3 and inhibit its expression and function in GBM cells, and ALOXE3 was significantly downregulated in human GBM and its knockdown in GBM cells promoted the growth of GBM and shortened the lifespan of mice (Li et al., 2021b). Other studies have found that FANCD2 and CD44 are significantly associated with the occurrence of GBM, TMZ resistance and GBM patient survival (Gulden et al., 2010). After the knockdown of TMEM161B-AS1, hsa-miR-27a-3p can downregulate the expression of FANCD2 and CD44, inhibit the proliferation, migration and invasion of U87 and U251 cells, promote cell apoptosis and ferroptosis, and downregulate lncRNA TMEM161B-AS1; moreover, overexpression of hsa-miR-27a-3p can downregulate the expression of FANCD2 and CD44 and inhibit glioblastoma growth in nude mice (Zhao et al., 2020; Jiang et al., 2021). The above findings are expected to provide promising therapeutic targets for the treatment of GBM (Sang et al., 2019).

### Crucial role of p53 in ferroptosis-mediated GBM

Overexpression of p62 in p53-mutant GBM promotes ferroptosis and suppresses SLC7A11 expression, whereas overexpression of p62 in p53-wild-type GBM attenuates ferroptosis and promotes SLC7A11 expression. p62 binds to p53 and inhibits its ubiquitination (Wang et al., 2018). The p53-NRF2 association and p53-mediated inhibition of NRF2 antioxidant activity are regulated by p62 according to the state of p53 (Zhang et al., 2020d). The dual regulation of ferroptosis by p62 requires the P53 mutational status (Jankowski and Rabinowitz, 2022; Song et al., 2022). In the p53-wild-type GBM, the classical p62-mediated NRF2 activation pathway plays a major role in regulating iron cell apoptosis, thus leading to increased SLC7A11 expression and acting as an anti-iron cell apoptosis mechanism (Ma et al., 2020). In p53-mutant GBM, the stronger interaction of mutant p53/NRF2 with p62 enhanced the inhibitory effect of mutant p53 on NRF2 signaling and reversed the classical p62-mediated NRF2 activation pathway, while p62 increased the transcriptional inhibition of SLC7A11 by p53, leading to the reduction of SLC7A11 and acting as a proapoptotic inducer. Taken together, the mutational status of p53 is an important factor in determining the response of GBM to p62-mediated targeted ferritin therapy (Hu et al., 2020; Zhu et al., 2021).

### Application of iron chelators in GBM

The application of iron chelators can block the cell cycle, usually at the G0/G1 phase, thus leading to apoptosis. Clinical studies have shown that iron chelators are potential new antitumor agents. A phase II trial of a single 5-day course of deferoxamine in 9 patients with neuroblastomas was performed in 1990. With 5 days of deferoxamine (an iron chelator) treatment, neuroblastoma regressions were observed in 7 of 9 patients at the dose of 150 mg/kg/day with an 8-h i.v. infusion daily. Meanwhile, the drug toxicity was not observed (Wang et al., 2021; Sun et al., 2022). However, there is no positive control drug in this study. Early studies of neuroblastoma cells and children with neuroblastoma showed that the iron chelator deferoxamine (DFO) has antiproliferative and antitumor effects (Yang et al., 2017). 89Zirconium-labelled girentuximab deferoxamine (89Zr-DFO-girentuximab) is on another clinical trial (NCT05563272). This is a prospective, open-label, phase II study to investigate PET/CT imaging in patients with solid tumors, including GBM patients with positive expression of carbonic anhydrase IX (CAIX). The primary purpose of this study is for diagnosis. This trial began on 3 October 2022. Followed by PET/CT scanning on Day 5 ± 2 days, the patients will receive single i.v. administration of 89Zr-DFO-girentuximab on Day 0. The researchers expected to enroll 100 participants of various solid tumors. They will evaluate the uptake of the 89Zr-girentuximab within the tumor deposits, and other outcomes, including heart rate, blood pressure, liver function, renal function and full blood count. However, the dosing of 89Zr-DFO-girentuximab is not released on the NCT official website (https://clinicaltrials.gov/ct2/show/study/NCT05563272). There are also some *in vitro* iron chelator studies on GBM cell lines. 10 μM deferasirox (DFX, a novel iron chelator) suppresses the proliferation of U251 and U87 GBM cells, induces cell cycle arrest in S phase and G2-M phase, and stimulates cytotoxicity and apoptosis (Legendre et al., 2016).

## Conclusion and perspectives

Extensive tumor heterogeneity is observed in glioblastoma, which can lead to a wide range of variable biological responses. Intratumoral molecular heterogeneity in glioblastoma is a major clinical challenge in terms of its recurrence and invasion. Intratumoral molecular heterogeneity in glioblastoma is twofold: on the one hand, it can be used as a prognostic marker to guide individualized treatment of GBM; on the other hand, it can be a factor in the failure of molecularly targeted therapy (Kong et al., 2011). The pathogenesis of GBM is not yet fully understood, and effective targeted therapies have not been developed. Therefore, elucidating the pathogenesis of GBM will facilitate the exploration of tumor blocking targets in a more targeted manner. Caution must be exercised in the use of iron chelators in the treatment of GBM. The question of whether the regulation of enzymes involved in iron metabolism is altered by GBM treatment (including radiation therapy and TMZ therapy) and whether these changes are effective in prolonging the survival of GBM patients remains to be addressed. Therefore, the application of iron chelators to GBM requires the exploration of appropriate treatment regimens to ensure that the desired therapeutic effect is achieved.

Furthermore, at present, the assessment for ferroptosis are limited to propidium iodide staining, transmission electron microscopy for morphological observation of shrinking mitochondria, and measurement of intracellular reactive oxygen species, 4-hydroxynonenal and malondialdehyde (Kong et al., 2021). No specific biomarker is available for ferroptosis. Therefore, with more in-depth investigations and solid evidences, GPX4 will be an effective marker for ferroptosis induced programmed cell death. Meanwhile, other components of ferroptosis signaling pathway, as Nrf2, ACSL4 and system Xc^−^, are also potential ferroptosis markers. Among them, system Xc^−^ (including SLC7A11 and SCL3A2), which are expressed at the cell membrane, are easier to be detected as a cell surface marker of cells undergoing ferroptosis. With more efforts from researchers worldwide, studies on ferroptosis markers and related drugs targeting the ferroptosis pathway will shed new light for the diagnosis and therapy of glioblastoma patients.
